# Effects of acute supplementation of *Panax ginseng* on endurance running in a hot & humid environment

**Published:** 2011-01

**Authors:** Fadzel Wong Chee Ping, Chen Chee Keong, Amit Bandyopadhyay

**Affiliations:** School of Education & Social Development, Univerisiti Malaysia Sabah, Sabah, Malaysia; * School of Medical Sciences, Universiti Sains Malaysia, Kelantan, Malaysia; ** Department of Physiology, University of Calcutta, University College of Science & Technology, Kolkata, India

**Keywords:** Acute supplementation, endurance, free fatty acid, heart rate, insulin, *Panax ginseng*, RPE, VO_2_

## Abstract

**Background & objectives:**

Athletes in Malaysia need to perform in a hot and humid environment due to the climatic nature of the country. c0 hronic supplementation of *Panax ginseng* (PG) (a deciduous perennial plant belonging to the *Araliaceae* family) enhances physical performance. As the ergogenic effect of acute supplementation of PG on endurance performance has not been explored in the Malaysian population especially in a hot and humid condition this study was taken up.

**Methods:**

Nine heat adapted recreational runners (age : 25.4 ± 6.9 yr, body mass : 57.6 ± 8.4 kg; body height : 168.3 ± 7.6 cm) were recruited in this placebo-controlled double-blind randomized study. Subjects ingested 200 mg of PG one hour before the exercise test on treadmill at 70 per cent of their VO_2max_ in a laboratory environment of 31 °C and 70 per cent relative humidity. They drank 3 ml/kg body weight of cool water every 20 min during the exercise to prevent adverse effects of dehydration. Blood samples were drawn every 20 min for the analysis of glucose, lactate, insulin and free fatty acids. Oxygen uptake was determined every 20 min while heart rate, body and skin temperatures, and ratings of perceived exertion (RPE) were recorded every 10 min during the trials.

**Results:**

Endurance running time to exhaustion did not differ between PG and placebo trials. Heart rate, skin temperature, core body temperature, oxygen uptake, RPE, plasma insulin, glucose, free fatty acid and lactate levels during the endurance exercise did not show any significant differences between the trials.

**Interpretation & conclusions:**

We conclude that acute supplementation of 200 mg of PG did not affect the endurance running performance of the heat-adapted male recreational runners in the heat.

Ginseng is used as a dietary and medicinal herb in different parts of the world. In the Western countries it is frequently used as a performance enhancer because it restores Qi, i.e., life energy^1^,^2^ *Panax ginseng* (Family - *Araliaceae*), often called as Asian or Korean ginseng, is the most widely used best grade of ginseng compared to the other species^2^. The root of this perennial herb has been used in oriental medicine since ancient times and it has become a popular tonic worldwide^3^,^4^. It has no teratogenic or mutagenic properties^5^. Ginseng is not included among the lists of banned stimulants for athletes^6^ because ingestion of *P. ginseng* did not result in positive test results for any banned substances after urine testing of elite athletes^7^. Accordingly, there is no risk of disqualification from drug-tested sporting events from using ginseng.

Numerous studies indicated that chronic supplementation of *P. ginseng* enhances the endurance performance, whereas some other studies demonstrated the contradictory results^8^–^10^. However, the effect of acute supplementation of *P. ginseng* on endurance performance has not yet been studied especially among Malaysians who are exposed to a hot and humid climatic condition throughout the year. The present study was, therefore, aimed to examine the effects of acute supplementation of *P. ginseng* on endurance performance in heat-adapted Malaysian male recreational runners under the hot and humid environment.

## Material & Methods

### 

#### Selection of subjects:

The study was conducted from April 2007 to March 2008. Nine male subjects aged between 20-40 yr of age were recruited in this study by random sampling method from the recreational runners [who jog at least 30 min, 2 times per week with at least a minimum of 30 min per session according to Jackson^11^ from the Malaysian students and staff of the Universiti Sains Malaysia, Kota Bharu, Kelantan, Malaysia. The subjects were evaluated in a randomized cross-over, placebo-controlled double blind method. Individuals with hypertension, asthma, diabetes, bronchitis, anaemia, cardiac problems, kidney or liver diseases and or any other major diseases were excluded from the study. The entire experimental protocol was explained to each subject before signing the consent form. The research protocol was approved by the Research and Human Ethics Committee of the Universiti Sains Malaysia.

#### Experimental design:

The subjects were evaluated in a randomized placebo-controlled double blind method. Each subject came to the laboratory for 5 times. The first three visits were spent for some preliminary tests whereas the final two visits were for the actual experimental trials (Fig. 1). The first pre-trial visit involved the measurement of sub-maximal oxygen consumption while second pre-trial visit included the measurement of VO_2max_. These two trials were conducted to determine the exact treadmill speed that corresponds to the 70 per cent of the subject’s VO_2max_which was set as the exercise trial speed. In the third pre-trial visit, subject was familiarized with the tests for experimental trials in the hot and humid environment (31°C, 70% relative humidity). Then, the two experimental trials with either PG or placebo (Pl) were carried out with a 7 days gap between trials. Subjects reported to the laboratory after an overnight fast of 10 hours. All the experimental trials were conducted at 0800 h.

**Fig.**
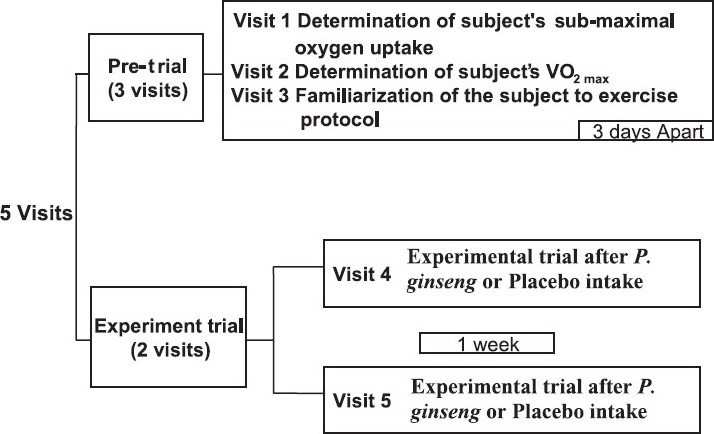
The experimental protocol of the study.

### 

#### Preparation of the subjects:

Subjects were requested to record their food intake for 3 days prior to the first trial and repeat the same diet over 3 days before the days of consecutive trials to minimize the variation in pre-exercise muscle glycogen status. Subjects were asked to refrain from heavy exercise for 24 h prior to the tests.

On arrival in the laboratory, the subject underwent a physical examination that included the measurement of body weight, pre-exercise heart rate and body fat percentage by bio-electrical impedance analysis (Tanita, Japan). After that, 4 skin thermistors (Yellow Springs instrument, USA) were attached to the chest, biceps, thigh and calf of the subject’s body for recording the mean skin temperature^12^. A rectal probe was inserted to a depth of 10 cm beyond the anal sphincter for the determination of core temperature of the body throughout the trial. Core and skin temperatures were recorded on a temperature monitor (Libra Medical ET 300R, USA).

A heart rate monitor (Sport Tester PE3000, Polar, Finland) was secured on the subject’s chest to monitor the heart rate. An indwelling cannula was inserted into a subcutaneous forearm vein and an extension tube was connected to it to facilitate repeated blood withdrawals. Patency of the cannula was maintained with heparinised saline. A standardized breakfast of 500 ml of water and a piece of bread was given to the subject 2 h before the trial. Subject ingested capsule containing *P. ginseng* (200 mg) or placebo one hour before the experimental trials^13^.

#### Procedure of submaximal exercise test:

After 5 min warm up at a treadmill speed of 6-7 km/h, subject was asked to wear a mouthpiece, a nose clip and heart rate sensor (Sport Tester PE3000, Polar, Finland). A head gear was fitted to support a two-way non-re-breathing valve (Hans Rudolph 2700 series, USA) attached to the mouthpiece. The gas collection was started when the pre-calibrated gas analyser system reached the steady state. The subjects ran on a motorised treadmill (Quiton 18-60, USA) for four minutes each at four different speeds (7, 8, 9 and 10 km/h, respectively). The speed was increased by 1 km/h at the end of each 4 min.

Expired air during the test was passed through a mixing chamber where sensors to the pre-calibrated paramagnetic oxygen and infrared carbon dioxide analyzers (Sensormedic 2900, USA) were used to determine the percentages of oxygen and carbon dioxide respectively in the expired air. Both the analysers were calibrated using two nitrogen based calibration gases (26% oxygen in nitrogen mixture, and 4% carbon dioxide and 16% oxygen in nitrogen mixture). The outputs from the gas analyser were processed using a computer for the calculation of oxygen consumption (VO_2_) and carbon dioxide production (VCO_2_). Expired gas was measured every 20 sec by the analyser. Heart rate and rate of perceived exertion were measured during the final minute of each 4 min of the increment.

#### Measurement of VO_2max_ :

Maximum oxygen uptake was determined using the modified Astrand protocol^14^ where subjects ran to volitional exhaustion during a continuous incremental run on a motorised treadmill. Subjects were initially allowed to warm up for 5 min at a low speed (6-7 km/h). After warm up, the subjects were fitted with the headgear, mouthpiece, nose clip and heart rate sensor as in the sub-maximal test. An appropriate speed (8-12 km/h) was selected and the test began with a grade of 0 per cent for 3 min. Thereafter, the grade was increased by 2Ɖ per cent every 2 min and the subject was encouraged to run until exhaustion.

Expired air samples were analysed at the end of each 2 min stage by following the same method as mentioned in the procedure of sub-maximal exercise procedure. Heart rate responses were also measured at the end of each 2 min stage. The maximum oxygen uptake (VO_2max_) value was accepted when the following criteria were met^15^:

- Attainment of a plateau in oxygen uptake despite increasing the workload.
- Heart rate within 10 beats/min of age-predicted maximum heart rate, i.e., 220 beats/min - age (yr).
- A respiratory exchange ratio of <gt; 1.15.

The first pre-trial visit was conducted to determine the correlation between oxygen uptake and running speed. The VO_2max_was determined in the second pre-trial visit. The data from these two trials were used to determine the running speed that would elicit 70 per cent of their respective VO_2max_during the experimental trials.

#### Experimental trial:

Subjects ran on the treadmill at 70 per cent of VO_2max_“to voluntary exhaustion” that was determined as the point when they indicated that they could no longer run at the required speed^16^. At the point of exhaustion and to ensure that the subjects were truly fatigued, the running speed was reduced to elicit 60 per cent VO_2max_for 2 min. Thereafter, the speed was returned to the prescribed speed (70% VO_2max_) and the subjects were encouraged to run as long as possible. Verbal encouragement was given to the exercising subjects to ensure their maximum effort.

#### Data collection during the experimental trials:

Blood samples were collected and oxygen consumption was measured at an interval of 20 min throughout the running trials. Heart rate, skin temperature, rectal temperature, room temperature, relative humidity and rate of perceived excretion (RPE) (by Borg’s Scale) were noted at an interval of 10 min. Cool water (4-8°C, 3ml per kg of body weight) was given to the subjects at an interval of 20 min to avoid any possible adverse effects of dehydration^17^.

#### Biochemical analysis of blood parameters:

Plasma glucose concentration was determined by the enzymatic oxidation method^18^. Enzyme-amplified chemiluminescent technology and commercially available immunoassay kits (Siemens Medical Solution Diagnostics, 2007) were used to perform immunoassays of serum insulin level. Plasma lactate was analysed using the YSI 1500 SPORT Lactate analyser (YSI incorporated, Yellow Spring, Ohio, United States).

#### Statistical analyses:

Statistical Programme for Social Sciences version 14.0 (SPSS Incorp, United States) was used for the statistical treatment of the data. All the parameters were expressed as mean and standard deviation (±SD). Shapiro-Wilk test was used to check the normality of the population^19^,^20^. Repeated measure ANOVA was used followed by Bonferroni post-hoc analysis to observe the significant difference at P>0.05 level. Paired-t test was used to compare the difference between means. Ratings of perceived exertion (RPE) were analysed using Wilcoxon signed rank test.

## Results

The body mass index and body fat percentage indicated that the subjects were under the normal category^21^. Mean VO_2max_ reflected that the subjects had good cardiorespiratory fitness^22^ (Table I).

**Table:** Physical characteristics of the subjects (n=9)

Age (yr)	25.4 ± 6.9
Body mass (kg)	57.6 ± 8.4
Height (cm)	168.3 ± 7.6
Body fat (%)	15.6 ± 2.8
Body mass index (BMI)	20.2 ± 1.9
Pre-exercise heart rate (beats min)	67 ± 5.7
Peak heart rate (beats/min)	197 ± 6.5
V_O2max_ (ml/kg/min)	51 ± 8.3
Values are mean ± SD	

There was no significant alteration in room temperature (31.0 ± 0.2 and 31.0 ± 0.1°C, respectively) and relative humidity (69.0 ± 1.2 and 68.0 ± 1.4%, respectively) between the two trials. Running time to exhaustion was not significantly different between PG and Pl trials (88 ± 19.5 and 84 ± 21.4 min, respectively).

Heart rate, oxygen consumption, skin and core body temperature significantly (*P*>0.001, P>0.01) increased over time in the same experimental trial compared to the respective resting values (Table II). However, no significant difference was observed in these parameters between the two trials. There was no significant main effect of supplements on oxygen uptake during the trials.

**Table II:** Values of different physiological parameters during the experimental trials (n = 9)

Parameters	Duration of exercise (min)									
		0	10	20	30	40	50	60	84	88
Heart rate (beats/min)	Pl	67 ± 5.7	157 ± 18.3**	162 ± 16.8**	165 ± 16.8**	167 ± 15.2**	169 ± 14.6**	170 ± 13.7**	172 ± 9.6**	
	PG	67 ± 4.3	156 ± 15.6**	160 ± 13.3**	164 ± 12.2**	166 ± 12.9**	166 ± 12.4**	169 ± 13.5**		175 ± 11.4**
VO_2_ (ml/kg/min)	Pl	7.3 ± 1.3	34.02 ± 5.4**		34.9 ± 5.1**		35.21 ± 5.3**		35.82 ± 5.0**	
	PG	7.2 ± 1.2	33.68 ± 4.7**		34.44 ± 4.7**		35.56 ± 5.3**			36.21 ± 4.2**
Core body temperature (ºC)	Pl	36.46 ± 0.3	37.06 ± 0.5**	37.55 ± 0.7**	37.96 ± 0.8**	38.31 ± 0.8**	38.42 ± 0.8**	38.56 ± 0.8**	39.18 ± 0.3**	
	PG	36.58 ± 0.4	37.06 ± 0.6**	37.52 ± 0.7**	38.02 ± 0.7**	38.27 ± 0.7**	38.35 ± 0.8**	38.6 ± 0.8**		39.3 ± 0.3**
Skin temperature (ºC)	Pl	33.04 ± 0.7	33.96 ± 0.7**	34.18 ± 0.6**	33.78 ± 0.8**	33.68 ± 0.9*	33.53 ± 0.8*	33.56 ± 0.8*	33.82 ± 0.7**	
	PG	33.21 ± 0.5	34.15 ± 0.6**	34.07 ± 0.5**	33.89 ± 0.7**	33.75 ± 0.7*	33.64 ± 0.7*	33.58 ± 0.6*		33.9 ± 0.6**

Values are expressed as mean +SD; PG, P. ginseng trial; Pl, Placebo trial P

P*<0.01

** <0.001 compared with the pre-exercise (0 min) value in the same trial

Plasma lactate, blood glucose, insulin and free fatty acid concentrations during rest and exercise trials are presented in Table III. Repeated measure ANOVA showed that there was a significant main effect of time on plasma lactate concentration (*P*>0.01) but there was no significant difference in the plasma lactate concentration between the trials.

**Table III:** Values of different blood parameters during the experimental trials (n = 9)

		Duration of exercise (min)					
Parameters		0	20	40	60	Time to exhaustion	
						Placebo trial (84 min)	Caffeine trial (88 min)
Plasma lactate (mmol/L)	Pl	1.52 ± 0.31	5.15 ± 2.19**	4.52 ± 2.42**	4.14 ± 2.04**	5.10 ± 0.68**	
	PG	1.47 ± 0.48	4.68 ± 2.17**	4.32 ± 2.05**	3.29 ± 2.19**		5.38 ± 0.81**
Plasma Glucose (mmol/L)	Pl	5.24 ± 0.7	5.47 ± 0.82**	6.28 ± 1.51**	5.87 ± 0.74*	5.77 ± 1.23*	
	PG	5.17 ± 0.59	5.32 ± 0.52**	5.70 ± 1.1**	5.72 ± 0.72*		5.36 ± 1.59**
Plasma insulin concentrations (*µlu*/ml)	Pl	7.32 ± 4.37	4.01 ± 2.24**	3.85 ± 2.01**	3.30 ± 1.72**	2.70 ± 2.1**	
	PG	7.0 ± -2.3	4.06 ± 2.71**	3.23 ± 1.81**	2.9 ± 1.59**		2.4 ± 2.0**
Plasma free fatty acid (mmol/l-1)	Pl	0.36 ± 0.25	0.42 ± 0.23**	0.49 ± 0.15**	0.69 ± 0.18**	0.95 ± 0.25**	
	PG	0.38 ± 0.18	0.41 ± 0.21**	0.54 ± 0.25**	0.74 ± 0.39**		1.01 ± 0.25**

Values are expressed as mean ±SD; PG, *P. ginseng trial*; Pl, placebo trial

P*<0.01

** <0.001 compared with the pre-exercise (0 min) value in the same trial.

Repeated measure ANOVA exhibited a significant (*P*>0.001, P>0.01) main effect of time on plasma glucose, insulin and free fatty acid concentrations. However, there was no significant variation in these parameters between the trials.

Subjects expressed their feelings on a numerical scale (RPE by Borg’s Scale) to indicate the fatigue level (Table IV). RPE increased significantly (*P*>0.05) with the progression of exercise in both the trials, but no significant variation was found in this parameter when compared between the two experimental trials.

**Table IV:** Rate of perceived exertion (RPE) of the subjects (n=9) during exercise in different trials

Supplement time (min)	*P. ginseng* trial	Placebo trial
10	10.7 ± 2.4	10.6 ± 2.4
20	11.8 ± 2.1	12.0 ± 2.3
30	12.8 ± 2.1	13.0 ± 2.6
40	13.6 ± 2.2	14.2 ± 2.5
50	14.8 ± 1.8	15.3 ± 2.7
60	16.0 ± 2.3	16.7 ± 2.6
End time to exhaustion	19.8 ± 0.8	19.6 ± 0.5*

Values are expressed as mean ± SD

* *P*<0.05 when compared with pre-exercise (0 min) value in the same trial

## Discussion

*P. ginseng* had long been used as an ergogenic herb for its beneficial psychophysiological effects which help to improve the endurance capacity, strength, neural functions, immune system and psychological parameters without any harmful effects to the body^5^,^23^.

Chronic supplementation of *P. ginseng* has been shown to augment the endurance performance^8^–^10^. However, other investigations revealed the contradictory findings^24^–^26^. The main finding in the present study was that a single dose of 200 mg of *P. ginseng* supplemented one hour prior to the experimental trial did not affect the endurance running performance and other selected physiological parameters in the heat.

However, other studies have shown that chronic supplementation of *P. ginseng* improves endurance performance and selected physiological parameters. For example, six to nine weeks of *P. ginseng* supplementation at a dose of 200 mg per day in male elite athletes significantly improved the oxygen uptake, endurance capacity, vital capacity, forced expiratory volumes, recovery heart rate, pectoral and quadriceps strength and reduced the lactate production in comparison with placebo group^9^. On the other hand, 8 wk supplementation of *P. ginseng* at a dose of 100 mg twice daily did not change the cardiorespiratory parameters as well as in the blood lactate level of healthy subjects^27^. Engels and Wirth^26^ supplemented healthy subjects with *P. ginseng* at a dose of 200 mg per day and reported that it did not induce any changes in cardiorespiratory parameters, blood lactate and RPE. In the present study, acute supplementation of *P. ginseng* with similar dose also did not exhibit any significant changes in the studied parameters.

Pre-exercise heart rate and working heart rate were not influenced by the acute supplementation of *P. ginseng*. This finding was similar with other studies that examined the effects of chronic supplementation of *P. ginseng* on endurance performance^26^. In the present investigation, the exercising heart rates increased significantly compared to the resting values at the end of both the exercise trials to meet increasing requirement of the body during physical exercise.

In both the exercise trials, oxygen uptake increased significantly compared to the corresponding resting values to meet the excess metabolic demands of the exercising muscles during the endurance running performances. However, oxygen uptake during the exercise did not show any significant difference between the two trials as also reported in other studies^28^.

No significant differences in core body temperature and in skin temperature was found during the exercise trials indicating that *P. ginseng* did not affect the body temperature regulation mechanism during endurance performance as also reported in a previous study^28^. However, in both the trials, skin and core body temperature were significantly higher during the exercising condition than the corresponding resting values.

A significant increase in plasma lactate concentration was noted during exercise in both the trials compared to the corresponding resting values. In both the trials, blood lactate started to increase from the onset till exhaustion. This could be due to either increased lactate production or its decreased clearance or combination of both. It may also be speculated that the increased lactate production is due to increased contribution of non-oxidative energy pathways imposed by insufficient blood flow to the exercising muscles.

Plasma free fatty acid is the most important energy source to sustain prolonged endurance activity. In the present investigation, fatty acid concentration increased from the onset of the exercise till the end time to exhaustion. There was a significant increase in plasma free fatty acid at the end of the exercise trial compared to their corresponding resting values in both the trials. Such steady increase indicated the availability of the substrate (fat) for its utilisation as the major energy source during the endurance running trials. PG trial reported no significant difference in plasma free fatty acid concentration in comparison with Pl trial during endurance performance as also reported earlier^26^. There was no significant difference in RPE between the two trials as also reported in previous investigations^26^.

The reason why there were no significant changes in the parameters measured might be attributed to the acute supplementation regimen of *P. ginseng* (1 h prior to the experimental trial). The significant effect of *P. ginseng* was observed when the supplementation period was longer. In addition, it was reported that its benefits was only best seen in poor physically-conditioned individuals^29^. Liang *et al*^30^ reported that in untrained adults, consumption of 1,350 mg P. *notoginseng* capsule per day for 30 days improved their endurance time more than 7 min during endurance cycle exercise. Hence, we postulate that the active ingredients in PG were not sufficiently absorbed during the trials to elicit any positive effects in our subjects who were recreational runners.

In conclusion, acute supplementation of 200 mg of *P. ginseng* one hour prior the exercise test did not impose any significant effect on the physiological parameters measured during the endurance running performance in healthy recreational runners in a hot and humid environment. Further research is needed to explore the effects of chronic supplementation of *P. ginseng* at higher doses on the running performances in a hot and humid environment.
